# Interview with Dr Jan Wise

**DOI:** 10.1192/bjb.2021.94

**Published:** 2022-02

**Authors:** Abdi Sanati

**Figure:**
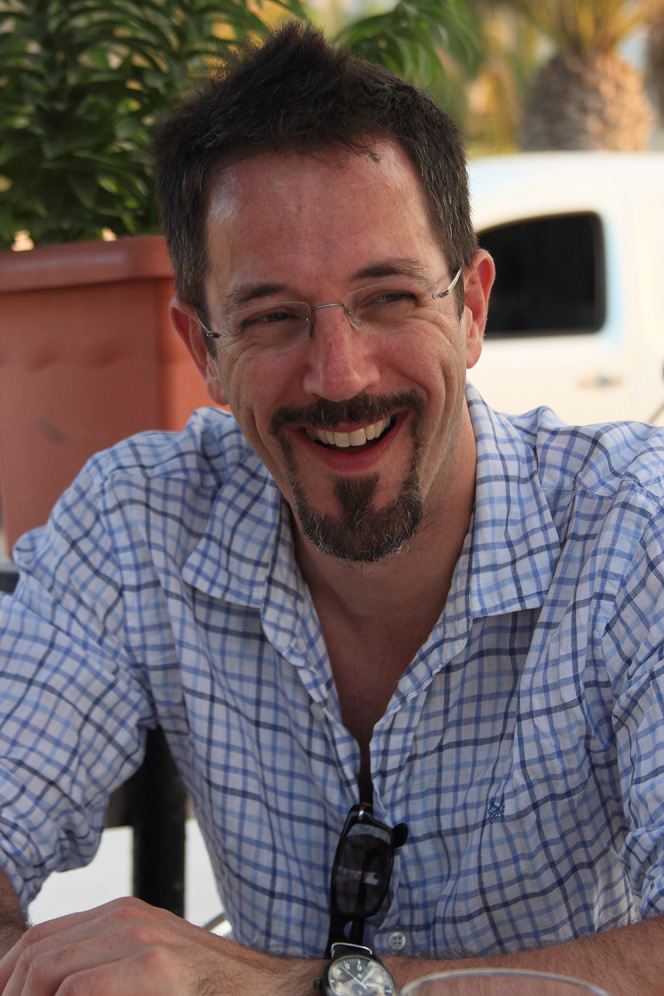

Dr Wise is a consultant in general adult psychiatry working in inner city London. I first met Dr Jan Wise around 15 years ago through another colleague. At that time, Dr Wise was already active in the British Medical Association (BMA). I found his knowledge of medicolegal and contractual issues vast and unparalleled. I have always admired his wisdom and his structured approach in solving problems. He remains active in the BMA and is currently the chair of BMA Medicolegal Committee. What made him stand out for me was his work on physician burnout, something I faced a few years ago. Dr Wise is also active at international level. He is elected to the Board of European Psychiatric Association. In addition to that he is also an Honorary Member of the World Psychiatric Association and Distinguished International Fellow of the American Psychiatric Association. I caught up with him to discuss issues such as pressure on psychiatrists and his international work.

**Thank you very much for agreeing to this interview Dr Wise. You mentioned in your article that going an extra mile endangers staff and patients. Could you elaborate on that statement please?**

We are a finite resource. We are human. We are limited and we are fallible. So, even under normal circumstances, there's a limit to how long we can stay on the board, clerking a patient, how long we can sit in casualty comforting the truly distressed psychotic patient who is ripping their hands to shreds, trying to escape the manacles the police have put them in. And this is a never-ending tide. There is always a need to care, there is no end to the misery that is out there, that we want to address. And if we do not place limits on ourselves, we will wear ourselves out. Even before COVID-19, we wore out faster than we were replaced. If I'm going to say that more succinctly, ‘We will never run out of patients, we will run out of you’. So staff have got to look after themselves, to avoid burnout, the moral distress that we're seeing at the moment. We hear from the BMA and in surveys that there are unprecedented numbers of doctors in general – not just psychiatrists – who want to leave, who want to reduce their hours, who don't want to do this.

**Thank you, another thing that comes up is the danger of being at risk of clinical negligence charges.**

Yes. People go into medicine because they want to help others, and that urge to help often clouds our judgement. Things are much better now than they were 25 years ago when some of us started. With exception reporting and reduced hours, we are less often in the scenario of people being so tired that they fall asleep at the nursing station. But what we forget is that the General Medical Council is going to hold you to the standards of the reasonable doctor with your experience. So if you're underperforming because you're excessively tired, whether that's from a long shift, having young children, or sick relatives that you are caring for, if you make a mistake, being tired is unlikely to be a defence. So, when people stay beyond their shift, or they have covered a night because the trainee was sick and there was no one to come in, we need to protect ourselves by letting the system know we are not fit to deliver care, because making an error by staying behind really puts your registration at risk, let alone the very unpleasant process of a relative or a patient deciding to sue you, because you made a mistake.

**Thank you. Reading your work on this topic is very interesting. It seems that going the extra mile has been celebrated as something of value, you're expected to say it in interviews that you are willing to go the extra mile. It is expected from us now.**

And that's the problem. If someone said they would never go the extra mile that would be a disaster for the profession and for the person. The Royal Army Medical Corps has as its motto *In Arduis Fidelis*, faithful in adversity. And in unusual circumstances, I would hope we would pull together for something like the Clapham rail disaster, Grenfell Tower or Piper Alpha, and we would all be there. But when it is not unusual, when it's not even every month, but it's every week, then it's wholly inappropriate to go even the extra 10 metres, because it just allows the government to underfund the service. And eventually people won't be able to go the extra distance and all patients will suffer, because we didn't say enough is enough.

**You have written about it in the context of the whole of profession of medicine. How do you specifically see it in psychiatry?**

Well the good news is, we have excellent training in boundaries, so it's relatively easy working in a profession where people aren't going to die if you don't do it, to have less porous boundaries than someone working in emergency medicine or oncology. I think the trainees are beginning to see from some consultants how to have an appropriate work–life balance. If they go the extra distance on Monday, they go short on Tuesday, Wednesday or Thursday, but on one day that week, they leave early to make up for the extra delivery. I think that is becoming easier. But what I hear, from colleagues and from other services like child and adolescent mental health services, are phrases like we need to get better at saying we are not commissioned to do that. Doing something that we are not commissioned to do, is to deliver it for free. Which means we are not delivering what we were commissioned to do unless we are delivering more hours than we should be. In a very concrete way, restricting yourself to what is commissioned in the time that is commissioned, and making it clear why you are doing that, helps managers argue for the funds they need. It is very simple for management to say it takes 12 man hours a day to deliver that and you have only commissioned 8 man hours, and many consultants are making it clear that they have used to give you 12 but they do not feel appreciated, so they are now only doing what you pay for.

**One thing I liked about your writing is emphasis on contracts, because we are contracted to deliver something, but many of us go beyond our contracts and feel it is a moral duty to do so.**

There are many different types of expectations. Everyone has an internal model of what they would like to be, different from what they are. One of the goals of therapy is to get people to marry those models because it leads to more happiness. The reason I mention that is, I would really hope that just as we say to an adolescent that peer pressure is not a good reason to give up your virtue prematurely or to take drugs, I don't see why peer pressure is a good reason to overdeliver, to stay late or start early. Now, as you've mentioned, there are other pressures, we have expectations on ourselves. If you're a perfectionist and everything has to be just so, it is a real struggle to make it perfect with the resources we have now. It was a real struggle 10 years ago, and we do not have now what we had 10 years ago.

**I was thinking that if the service is well-functioning, does it need people to work beyond their contract?**

It very much depends upon how one defines well-functioning. Deming, an economist, said that every system is perfectly designed to get that to give the results it gets. If the staff are working to their contracts then they are not overdelivering. If that delivers what was commissioned then that is ‘well-functioning’ – but it may not be a ‘good service’. But I am not sure we will ever have an average service that achieves that because, on average, consultants across the UK are working 4–6 hours a week beyond what they're contracted to do.

**Another issue is physician burnout, as you mentioned doctors are burning out. And one thing that I fear is that in the post-pandemic National Health Service (NHS), we will have even more and more burnout. I do not think giving more money would fix it.**

In the short term, the pandemic has been absolutely horrendous. It's been horrendous for its acute devastation, but also because it has eroded rest and recreation. People cannot travel, and rest and recreation is more than just not being at work. It is seeing our loved ones, seeing friends and family, going and doing those interesting things that feed the soul and spirit. There are also positives from the pandemic. We have learned that we don't need endless meetings, or to be in the same room for procedural issues. It kills creativity.

You can't do research development across Zoom. Unless you already have a really strong relationship with your peers, it's very difficult for those ideas to zing around the room to snowball and become something truly exhilarating and exciting. So we do need in-person meetings, but we are no longer losing hours to get to a room that we feel obliged to sit in to listen to something for 45 min when we really need to only hear 10 min. That actually is great for democracy like medical staff committees or local negotiating committees, because it means that you can do them more often. So the time before a response has been given is dramatically cut. You can have your consultant body complaining about the on-call rota or raising issues about trainees safety or highlighting that there's a personality conflict with a clinical director much faster than ever before. So I agree that it is exhausting, that everybody is tired, but it has given some real opportunities for change, including the realisation that there's got to be more to life than work.

**That is very true. Staying on the issue of burnout, I remember that in a conference some leading scholars put the responsibility of burnout on the individual. If the individual walks away they wouldn't burn out. What about the role of the system?**

I think you are referring to the sense that resilience in a way, blames the person for not being able to cope. And it is very difficult to build resilience in a disaster. The NHS does not have enough resources, if there were more staff, more time, less demand, we wouldn't be burning out like this. If you think about burnout as the consequence of friction between personal aspiration and what can be delivered, we know that the NHS is like a supertanker, it takes years to change its directions. It takes over a decade to train a new consultant. So, if we want to keep on doing things the way we're doing them, people are going to burn out, we will fail. We should be creative, for instance using nurse prescribers, or physician assistants, changing our view about handling risk. There are tasks that can be dropped or allocated to rapidly trainable members of staff that would change their day-to-day jobs. None of us do the mundane activities we did 20 years ago, or even 10 years ago. They've been devolved to other staff. I'm concerned that when we take back some of those administrative duties such as booking or changing appointments, even with an app, it is inefficient. So it's helping people to think about why does it feel useless, what is the frustration and how does one fit within that? That, I think, is the key to maintaining longevity in the service, which is critical in a way it wasn't previously; people are now going to be in psychiatry till 67 years of age, probably 69 for those who are starting now, because the state pension age will go up, rather than leaving at 55 with mental health officer status.

**Do you think the mental health officer status should be reinstated?**

Yes, from a pragmatic perspective. However, if they do not change the pension rules, then when people hit 50 years old or thereabouts, they will need to go part time, which is a good thing. We have a paucity of child and adolescent services in north-west London. There are charities interested in growing the independent sector provision, and they were very, very concerned that this would harm the NHS. A couple of us spoke with them, pointed out the waiting lists and the barriers to providing more. And it helped them understand that were they to point out to relatively new consultants that they can work in the independent sector for part of the time, in a multidisciplinary team, they will actually grow capacity to treat an underserved proportion of the population. There are also those who've retired with mental health officer status who are available, and there will be those who need to reduce their provision to the NHS because of breaches of the annual allowance, who are also available. So independent provision is not necessarily at the detriment of NHS provision, it can be positive for patients and positive for staff.

**It's interesting because NHS and the private sector are presented as the polar opposites, and private is seen as kind of the dark side. I think a healthy private service could help the NHS.**

Absolutely. If people have a reasonable alternate source of income they're not beholden to a single master, they're able to point out inappropriate working conditions, lack of respect or the shameful physical environment. Why should patients and staff have to put up with substandard facilities? There is no reason why we shouldn't support independent provision, which is not in direct conflict with the NHS, e.g. the independent sector can't really provide adequately for psychosis. Although I do hear a growing view that the NHS can't provide adequate care in metropolitan areas.

**One think I have witnessed is the constant reorganisation of the services, and what do you think is the effect is on psychiatrists?**

Well one of the effects is understanding it's a life cycle event. The first time it happens, you're full of enthusiasm, it's going to solve these problems. The second time, well, maybe they just didn't do it right the first time. The fifth time, your main concern becomes, how am I going to get a solo office. Will I still have a secretary? One's enthusiasm for the delivery of clinical care can erode with time, I exaggerate for dramatic purposes. In the last reorganisation we had, I delivered a piece of work pointing out that the manpower calculations for the metrics that were planned meant that staff would have no breaks in a week. This was nursing staff, so that was inappropriate. The revised metrics still meant that they only got half an hour's break in the week! So, consultants still have an important role in reorganisation. But we need to bear in mind that the primary purpose is often not what we're told this is for. It is politically driven, it hides budget or manpower cuts. Consultation must be meaningful; to paraphrase, no change to me, without me!

**And how do you see the increase in bureaucracy in psychiatry? That is one of my pet hates, I have to admit.**

It goes hand in hand with an increasing conviction that if you measure everything, you can prevent the things you want to prevent. One of the biggest drawbacks I found of electronic records is the loss of the narrative. I am fully aware that this may be harking back to an inglorious past that was never present. But when I look at case files for medicolegal reports, I see the old discharge summaries, and someone's life is explained in glorious detail. It's very difficult to piece that together nowadays from an electronic patient record. There are advantages, one can cut and paste the highlights quite easily. But it then appears to be that all one is reading is cut and paste, for the large part.

**One thing that I always ask about is clustering, and the way patients are reduced to clusters.**

Yes. Some people have gone as far as identifying clusters with diagnosis. And how is that going to help them when we start using ICD-11!

**It's interesting you mentioned narrative. One thing, as psychiatrists, we used to be trained in, was psychopathology, which emphasises narrative. And it's kind of being pushed out of the curriculum. And that is, I think, to the detriment of psychiatry. What do you think?**

It is sometimes surprising the lack of scepticism that I see in trainees. Why is this patient presenting in this way at this time? It's relatively easy to say why in this way, but there's not enough attention to why at this time. We underrecognise the degree of secondary gain that can be present. Whether it's problems with neighbours, benefits or family. Often, that is the secret to understanding why there's a deterioration now.

**You are very involved with European psychiatry. Reading some articles from mainland Europe, I think there is a gap between UK and European psychiatry, and we need to actually get more together.**

Definitely. One of the things that's very odd about looking at other systems is, it's really easy to idolise them or denigrate them. So for years, I was going to the American Psychiatric Association for clinical excellence and the European Psychiatric Association for connections, networking and friendship. As time went on I realised that the USA does have truly outstanding Centres of Excellence. But the average UK psychiatrist, in my view, was better than the average USA psychiatrist. Looking at Europe, what came across was the importance of cultural differences and local circumstances. You do need unique solutions for different places. In Iceland in the middle of winter, when you can barely travel to the next building because of the snow and the wind and the ice, meant they were world leaders in telepsychiatry 30 years ago. And then you compare that with Denmark, who had a huge influx of refugees during the Yugoslav War, but a tremendous shortage of translators, they started using telepsychiatry for interpreters for psychological treatments for post-traumatic stress disorder. There are different approaches to hospitalisations, so Italy has a very low rate of compulsory hospital admissions compared with the UK. It's being exposed to these that leads you to see important clinical differences that arise from legal and political differences. It also highlights what can go horribly wrong if you don't pay attention to the politics.

**I think politics also manifests itself in defensive practice. The fear of going to Coroner's Court. It's something that worries me that we will be more defensive, and it doesn't serve patients well.**

It is an attempt to protect oneself against an unknowable risk. As Professor Wasserman has stated, one can reduce suicide at a population level, but not at an individual level. If you very thoroughly treat every single patient, the same number are probably still going to die, but for most people, that is so horrible that they can't run with it. One of the things I've noticed is by accepting that there is a risk that cannot be eliminated, and once one's done what one can do, and been clear about therapeutic risk, there are usually fewer adverse outcomes. Partly because you've put the risk on the table and said we've done what we can do, that there is a chance it will go horribly wrong, but if we don't take this risk it will never get better, or by being clear the risk is not one that psychiatry can solve, or is commissioned to solve.
